# A review on introduced *Cichla* spp. and emerging concerns

**DOI:** 10.1016/j.heliyon.2020.e05370

**Published:** 2020-11-04

**Authors:** Shantika Maylana Sastraprawira, Iqbal Harith Abd. Razak, Salwa Shahimi, Siddhartha Pati, Hisham Atan Edinur, Akbar Bavajohn John, Amirrudin Ahmad, Jayaraj Vijaya Kumaran, Melissa Beata Martin, Ju Lian Chong, Ahmed Jalal Khan Chowdhury, Bryan Raveen Nelson

**Affiliations:** a Institute of Tropical Biodiversity and Sustainable Development, Universiti Malaysia Terengganu, 21030 Kuala Nerus, Terengganu, Malaysia; b Faculty of Marine and Environmnetal Sciences, Universiti Malaysia Terengganu, 21030 Kuala Nerus, Terengganu, Malaysia; c Research Division, Association of Biodiversity Conservation and Research, Devine Colony, 756001 Balasore, Odisha, India; d Forensic Science Programme, School of Health Sciences, Universiti Sains Malaysia, 16150 Kubang Kerian, Kelantan, Malaysia; e Environmental Futures Research Institute, Griffith University, Nathan, Queensland 4111, Australia; f Institute of Oceanography and Maritime Studies, Kulliyyah of Science, Jalan Sultan Ahmad Shah, 25200 Kuantan, Pahang, Malaysia; g Centre of Excellence for Entrepreneurship Research and Innovation, Universiti Malaysia Kelantan, Locked Bag 36, Pengkalan Chepa, 16100 Kota Bharu, Kelantan, Malaysia; h Department of Marine Science, Kulliyyah of Science, International Islamic University Malaysia Kuantan, Jalan Sultan Ahmad Shah, 25200, Kuantan, Malaysia

**Keywords:** Cichlid, Peacock bass, Fisheries, Management, Ecology, Invasive, Animal physiology, Biodiversity, Ecosystem services, Environmental risk assessment, Nature conservation

## Abstract

Peacock bass (*Cichla* spp.) originates from the Neotropical environments of Brazil and Venezuela but, through trade and smuggling for aquarium keeping, sport fishing and aquaculture, it is now an emerging concern. Yet, less is known for *Cichla* spp. distribution and its ability to invade new environments. Aimed to communicate on *Cichla* spp. ecology, biology and introduction schemes from Scopus, Web of Science, Google Scholar and also National Centre for Biotechnology Information, this review also contains management strategies for invading fish species. While *Cichla* spp. can displace native fish populations, this concern is explained using ecological functions, physiological demands, direct and secondary invasion, disease tolerance and parasite spillover. Briefly, *Cichla* spp. has rapid embryogenesis (72 h) and matures in short periods (11–12 months), giving it an advantage to colonize new environments. With a large appetite, this true piscivore gains territorial control over water bodies by making it their feeding and nursery grounds. Perceived as an emerging concern after becoming introduced, seal-off or sport fishing were used to manage *Cichla* spp. but, this practice is not sustainable for the entire ecosystem. Hence, we recommend bottom-up management that involves community participation because they interact with the fish and have knowledge about their environment.

## 1 Introduction

South America is a notable Neotropical biodiversity hotspot in which large mouth bass, silver croaker, golden dorado and peacock bass (*Cichla* spp.) are endemic. These fish were involved in pet trade, game fishing and aquaculture and therefore, have become emerging concerns in areas they were introduced (Willis et al., 2015; Bower et al., 2016; Doherty et al., 2016; McGeoch et al., 2016; Seebens et al., 2017; Bezerra et al., 2019). Considering freshwater ecosystems to have different current strengths, temperature and depths, it is the interconnected confluence, reservoir, watershed and meanders that determines the type of inhabitants for this environment (Liew et al., 2016; Franco et al., 2018). In the new environments, non-native fish like *Cichla* spp. easily adapt and dominate other species by becoming a predator (Agostinho et al., 2005; Liew et al., 2016). Their ability to easily invade an environment is knowledge that needs exploring.

*Cichla* spp. is recognized as a fish with large body, occupies freshwater environments, preys on other fish (piscivore), has distinct body markings and is endemic to Brazil and Venezuela. Researchers around the world consider *Cichla* spp. as a voracious predator that hunts and swallows their prey entirely (Hansson et al., 1998; Pace et al., 1999; Carpenter et al., 2010; Ellis et al., 2011). Researchers also observed that *Cichla* spp. predates on Characiformes, Osteoglossiformes, Siluriformes, Gymnotiformes and Cichliformes that measure 30–40 cm which means, this fish selects weaker prey that cannot outrun its chase (Zaret and Paine, 1973; Jepsen et al., 1997; Neal et al., 1999, 2006, 2017; Fugi et al., 2008; Aguiar-Santos, 2018).

The discovery of *C. temensis, C. ocellaris, C. monoculus,* and *C. kelberi* during 1960s favoured them for aquaculture produce, game fishing, pet and exotic species trade in Europe, North and Central America and Asia (Winemiller et al., 1997, 2001; Fugi et al., 2008; Pelicice and Agostinho, 2009; Espinola et al., 2014; Franco et al., 2018). It is possible that flash floods and damming have allowed fish to migrate between unconnected water bodies and inaccessible areas in Brazil, Peru and Venezuela (Zaret and Paine, 1973; Latini and Petrere Jr, 2004; Espinola et al., 2014; Agostinho et al., 2018; Franco et al., 2018). Therefore, in the course of 25 years, 15 species of *Cichla* were being discovered in South America (Vasquez and Rogers, 1992; Jepsen et al., 1997; Winemiller et al., 1997; Kullander and Ferreira, 2006; Willis et al., 2012; Mourāo et al., 2017).

Separately, some *Cichla* populations may possess closely linked ancestral alleles and this associated them to claims of natural hybridization (Feldberg et al., 2003; Neves et al., 2004; Arnold and Martin, 2009; Willis et al., 2010; Ellis et al., 2011; Willis et al., 2012). A hydrid with dissimilar DNA barcode is produced after same-species fish from different locations are confined together (Hubbs, 1995; Teixera and Oliveira, 2005; Jaffar et al., 2019). For instance, when both, ‘*Cichla temensis* and *C. monoculus*’ or ‘*C. temensis* and *C. piquiti*’ interact in a closure, their filials become recombinant hybrids (Feldberg et al., 2003; Neves et al., 2004; Teixera and Oliveira, 2005; Oliveira et al., 2006; Willis et al., 2015; Mourāo et al., 2017) and like other fish, lose their ancestral information (Telechea, 2009; Kuchta et al., 2014; Jaffar et al., 2019). Therefore, the sole reliance on molecular identification could wrongly identify a fish species.

**Figure 1:**
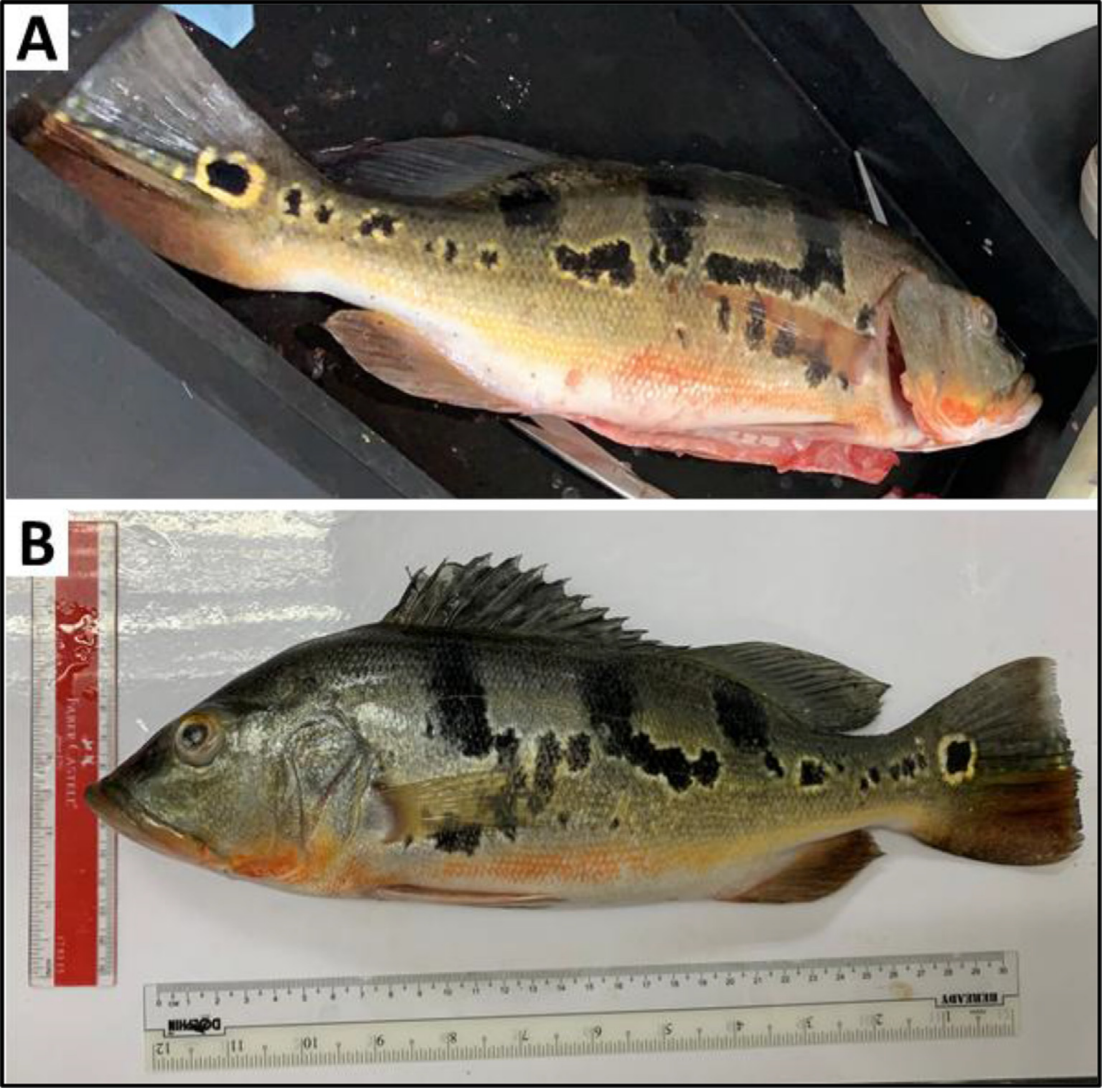
Peacock bass from Tasik Telabak (Malaysia) having bands and blotches of *C. kelberi* in both, (A) Right- and (B) Left-angles but, having coloration marks of *C. temensis*.

Identification for *Cichla* spp. should have the combination of morphometric and meristic indicators like shape, form and size and baseline information are available in Portuguese, Spanish and English (Oliveira et al., 2006; Marques et al., 2016). However, in a recent scenario, the *Cichla* sp. from Tasik Telabak (Malaysia) were having bands and blotches of *C. kelberi*, coloration of *C. temensis* (Figure 1) and posession of *C. kelberi* identity (via mitochondrial DNA – CO1; 92 % similarity index). At present, the argument is less conclusive because of sample size. Therefore, with an aim that focuses on history, biology and ecology (in the context of production, distribution, consumption and trade), introduced *Cichla* spp. is communicated as an emerging concern. This review also contains sustainable measures for (but, not limited to) invading fish species. Overall, exploring the social ethology and behaviour change of introduced fish allows researchers to predict the possibility of emerging concerns and plan for timely interventions.

## 2 Methodology

The inclusion and exclusion procedure followed protocols of John et al. (2018a, b) and Nelson et al., 2019a, Nelson et al., 2019b where keywords from the primary associations are used independently or added together (in combination of two or more) with activities, aspects and region (Table 1). These keywords revealed several documents in Scopus (n = 64), Web of Science (n = 71), Google Scholar (n = 103) and National Centre for Biotechnology Information (n = 1262). After the exclusion criteria that removed duplicated words, untraceable articles or invalid citations, a total of 128 unique and reliable sources were compiled. With all information restricted to peacock bass, native and global occurences and its biology, another line of exclusion criteria limits the age of information from January 1950 to February 2020. After the second layer of filtering, the final library contains information on peacock bass biology (origin, distribution and feeding), development (reproduction, embryogenesis and growth), introduction schemes (attraction, aquaculture, ornament, food, pet and angling), implications and challenges associated to species with emerging concerns and also relevant policies. Separately, strategies are provided after learning from strengths and weaknesses in previous attempts (Table 2). The protocols to device the new strategies were adopted from Agostinho et al. (2007) by considering climate and ecological conditions and also the trial and error experimental designs. With this, the suggested strategies are practical for invading fish species regardless the introduction scheme (aquaculture, pet trade or sport fishing) and their location.

**Table 1:** Keywords used to construct the information library on peacock bass.

Primary association	Region	Activities	Aspects
Alien Acanthocephala Annotation Characteristics Cichla sp. Cichlids Digenea Eradicate Fish species Gussevia aloides Invaders Invasive Monogenea Parasite(s) Peacock bass Piranha Predation Radiation Schistosome Tilapia Widespread	Asia Africa America Indonesia Lake Gatun Lake Victoria Malaysia Mexico North America Panama Poland Puerto Rico Rosana River South America Venezuela	Angler(s) Aquaculture Commercial fishing Livestock(ing) Ornament(al) Pet Recreational fishing Sport-fishing Evaluation Proposed	Aquarium fish Floodplains Freshwater Lake Open waters Commercial trade Conflict Conflict management Control Fish trade Law Management Pesticide

**Table 2:** Word strings related to non-native fish species management.

Concerns	Limitations	Source
Non-native fish species terminology	Biasness to understand further studies	Humair et al. (2014)
Biosecurity and awareness	Lack of responsibility and prevention led to introduction of non-native species; or released to increase wild stock diversity	Caffrey et al. (2014)
Species in inaccessible areas	The distribution of non-native fish species from the rarely considered source of introduction (e.g. sport-fishery)	Caffrey et al. (2014)
Introduction of non-native fish species	Low-density invasion is neglected, and becomes a significant problem which is arduous to elucidate	Schofield et al. (2019)
Cost analysis for non-native species management	Loses from management or impacts that are rarely considered	Caffrey et al. (2014)
Non-native species management	The old-fashioned method is used at compensation of time and cost	Boets et al., 2019
Conflict of interest	Government decision to increase local economy without considering environment impacts	Neal et al. (2017)

## 3 Results and discussions

### 3.1 Biology and ecology of *Cichla* spp.

**Figure 2:**
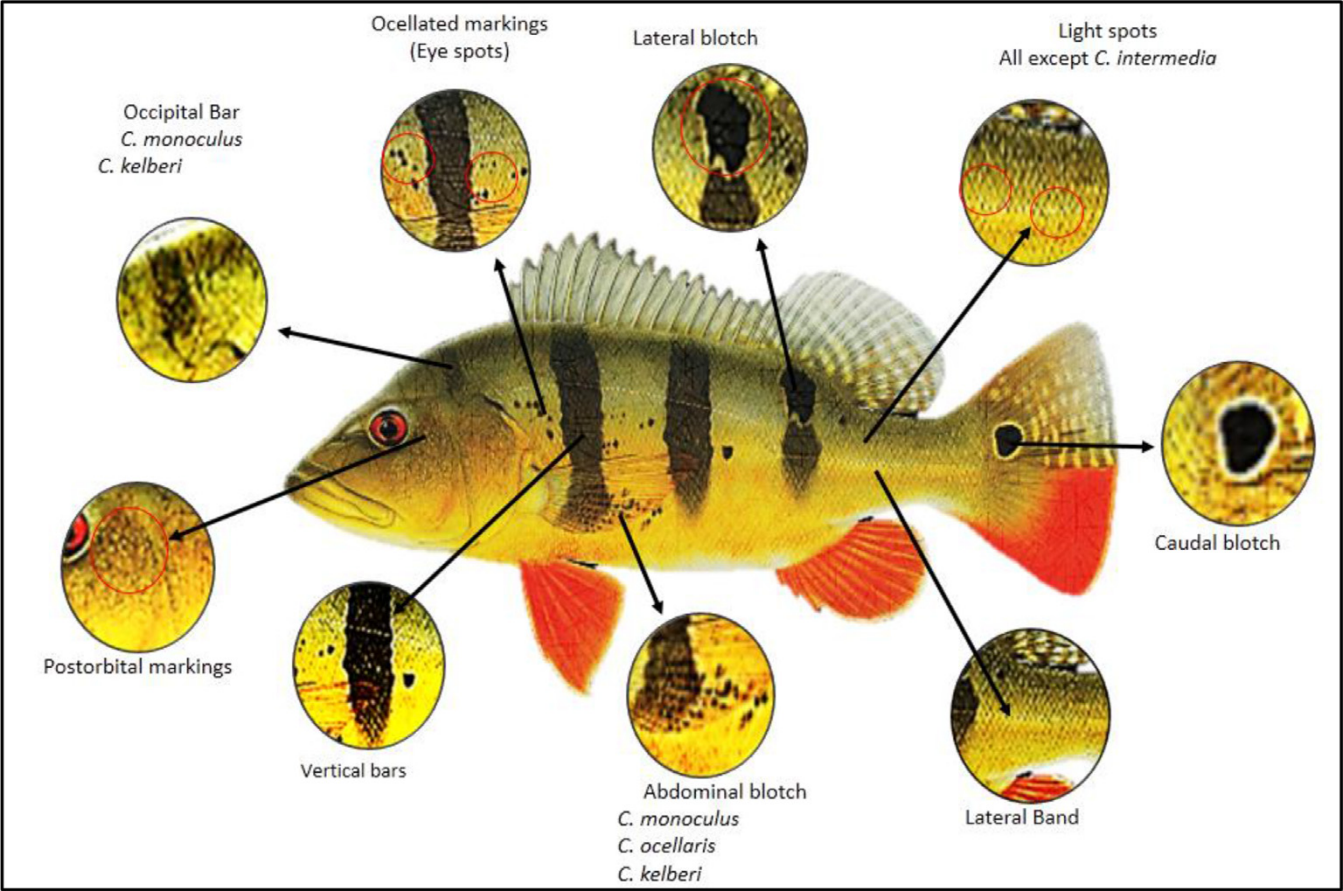
Distinct morphology markers to distinguish *Cichla* sub-groups.

A total of 15 *Cichla* spp. were identified having a bass-like shape and colourful body patterns (Zaret and Paine, 1973; Fugi et al., 2008; Vieira et al., 2009; Ota et al., 2019). Three vertical black bars (except *C. pleiozona* - four and *C. piquiti* - five) and black-encircled gold annulus are general morphology markers for *Cichla* spp. aside from the red colour at centre of the caudal keel that appears distinct to certain species (Figure 2). With four different body colourations, *Cichla* spp. are divided into yellow-gold (*C. jariina*, *C. pinima*, *C. kelberi*, *C. orinocensis*, *C.ocellaris*, *C. monoculus*, *C. vazzoleri*, *C.thyrorus*, *C. nigromaculata*, *C. pleiozona*), brown-green (*C. intermedia*, *C. temensis (A)*, *C. mirianae*), light grey (*C. temensis (P)*, *C. piquiti*) and blue-green (*C. melaniae*) variations.

In the native habitat, *Cichla* spp. has a size range of 30–60 cm, weighs 3–6 kg when matured and can survive up to 69 years (Jepsen et al., 1999; de Souza et al., 2011; Willis et al., 2012). It usually occupies deep water (10–20 m) with rock or submerged tree beds and spreads in 100–1000 m radius depending on the size of the water body (Hoeinghaus et al., 2003; Januario et al., 2019). In the Neotropical habitat, *Cichla* spp. resides in waters with 23–28 °C temperature, pH of 7.8 and dissolved oxygen concentration of ±5.0 mg l^−1^ (Fugi et al., 2008; Kovalenko et al., 2010; de Souza et al., 2011; Espinola et al., 2014; Franco et al., 2018).

After hatching, *Cichla* spp. begins its life cycle as a free-swimming larvae, juvenile and sub-adult before maturing in the adult form within 11–12 months. Across every stage of development, the cichlid preys on slow growing, weak and less competitive organisms (Zaret and Paine, 1973; Chapleau et al., 1997; Carpenter et al., 2010). For instance, two-day-old *Cichla* larvae predates on developing crustaceans and rotifers (Zaret, 1980; Winemiller, 1997), the juvenile feeds on insects, shrimps, and atherinids (Zaret 1980; Winemiller, 1997; Jepsen et al., 1997) whereas, adult *Cichla* spp. feeds on fish like poecilids, characids, eleotrids and cichlids with some instances of canibalism (Shafland, 1999a; Hill et al., 2005; Neal et al., 2017; Pereira et al., 2017; Sales et al., 2018; Bajer et al., 2019; Bezzera et al., 2019; Golani et al., 2019; Santos et al., 2019).

The spawning season of *Cichla* spp. occurs between October and May before the Neotropical raining season (Jepsen et al., 1999; Chellappa et al., 2003a; Holley et al., 2008). *Cichla* spp. displays an unspectacular or a gradual courtship because the male fish usually matures earlier than the female fish (Zaret, 1980). This cichlid has a homogeneous pair of gonads with interconnecting blood vessels that change colour and increase in size (sometimes reaching the swim bladder compartment) to demarcate maturation (Chellappa et al., 2003a; de Souza et al., 2011). The gonad maturation of a female *Cichla* spp. is described in several stages where during stage-1, a small nucleus develops in the somatic cell cytoplasm. Stage-II is reached when the somatic cell cytoplasm appears basophilic. Meanwhile, during stage-III, the somatic cell nucleus are surrounded by cortical alveoli and by then, the number of oocytes become plenty in both ovaries. Stage-IV is reached when somatic cell nucleus becomes almost transparent and during stage-V, the nucleus of somatic cells are ready to exude from the follicle.

*Cichla* spp. adopts an oviparous spawning method where fertilization takes place in the external environment. The female fish lays her eggs inside a flat depression dug by both brooders. Simultaneously, the male fish swims behind the female *Cichla* spp. and releases a cloud of sperm that settles and infuses the eggs. Parental guarding of eggs and hatchlings occur by taking turns, with both adults fasting intermittently but, the male *Cichla* spp. is seen more devoted than the female fish (Zaret, 1980; Murray, 2001). Embryogenesis completes within 72 h and follows with the hatching of free-swimming larvae (Zaret, 1980). Since adult *Cichla* spp. monitors the feeding frequency of hatchlings, the entire brood would migrate around the water body so that the growing larvae consumes a variety of prey. Parental guarding ends after their offspring develop intense body coloration (secondary sexual characteristics) which also includes the appearance of a nuchal hump (fatty tissue deposit) on the forehead of male *Cichla* spp. (Zaret, 1980; Jepsen et al., 1999).

### 3.2 Introduction schemes for *Cichla* spp.

#### 3.2.1 Ornamental attraction

Peacock bass are unified by their colour and body patterns which give rise to names like tucunaré-Aҫu (Portuguese, Brazil), lukanani (Hawaii), and pavón (Spanish speaking countries), while butterfly peacock, eyespot cichlid and peacock bass are general English references for *Cichla* spp. (Reis et al., 2003; Reiss et al., 2012). Most *Cichla* spp. are familiarized by their yellow gold body and grey-greyish sorrel abdomen aside from the three, four or five black vertical block-like shaped bars (Reiss et al., 2012). Aside from vibrant appearances, ease to acclimatize, minimal maintenance, aggression during angling and reasonable purchase value are the additional attractions of *Cichla* spp. to local communities (Liang et al., 2006; Liew et al., 2012). In fact, the vibrant colours and actively-moving nature of *Cichla* spp. symbolize energy and good fortune which perhaps, became reason for its involvement in the Asian ornamental fish trade (Edwards and Beck, 2002; Magalhāes et al., 2017). Hobbyists were unaware about *Cichla* spp. growth and demanding maintenance and therefore, illegal aquarium dumping was sought by irresponsible individuals (Duggan et al., 2006; Gertzen et al., 2008; Holmberg et al., 2015; Maceda-Veiga et al., 2016; Magalhāes et al., 2017, Figure 3). It only takes one fertile *Cichla* spp. brooder to invade a water body. For instance, undocumented release of *Cichla kelberi* through aquarium dumping led to a population burst (>60 % yield during single catch) in Lake Keneret (Israel) over the course of 22 years because it is deep, has oxygenic waters and contains sufficient food sources (Golani et al., 2019).

**Figure 3:**
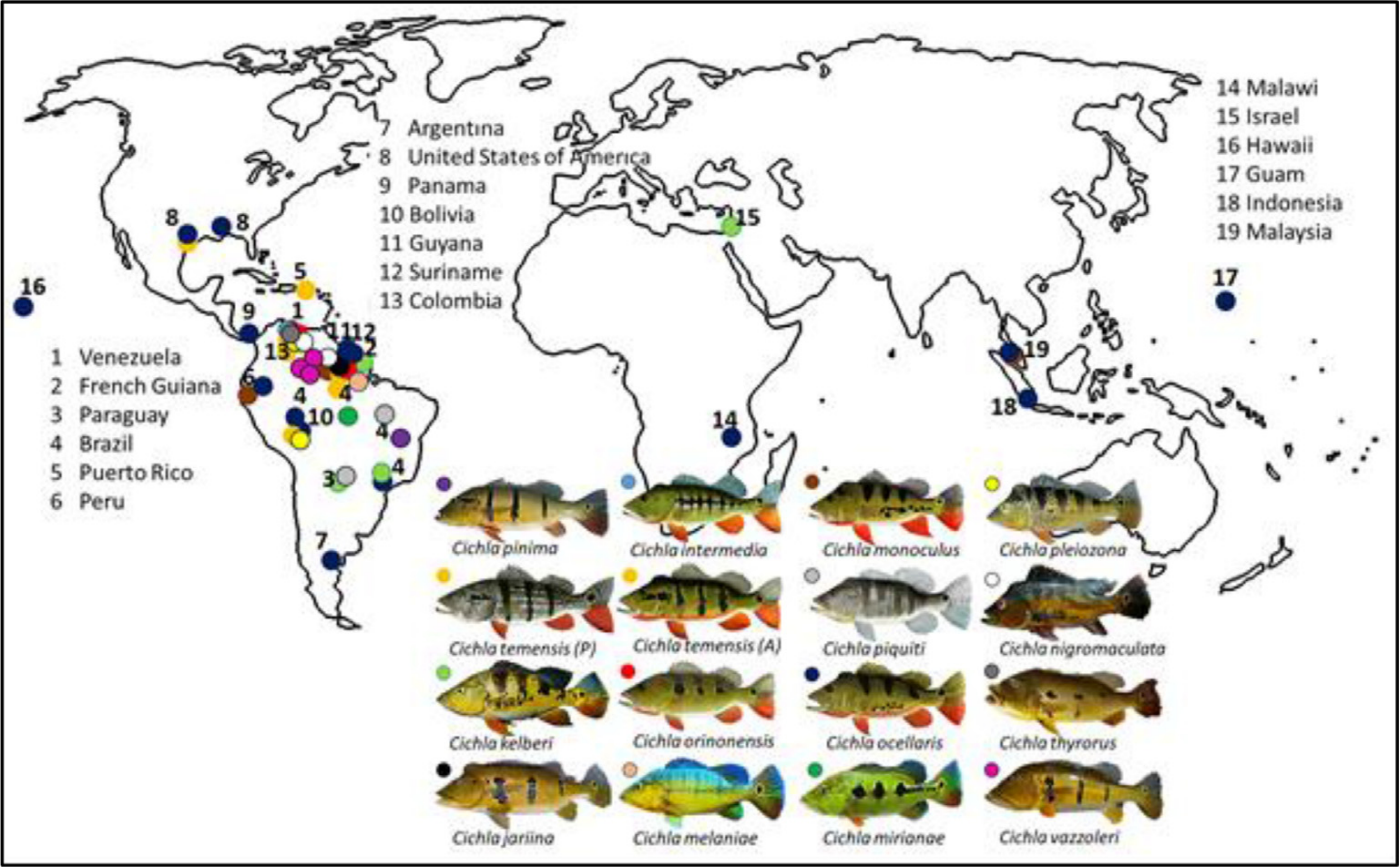
The distribution of *Cichla* spp. in North and South America, Middle East, Africa and Asia.

#### 3.2.2 Damming activities

Freshwater ecosystems are classified into tributaries, conglomeration and alluvial networks that have different flow energy and this feature defines the placement of damming projects that will swell rivers into man-made reservoirs (Song et al., 2019). Dams distribute biodiversity into oligotrophic (before the dam) and eutrophic sections. Therefore, authorities are blamed for decisions to release various types of fish into oligotrophic waters. In fact, authorities perceive such fish to have aesthetic values that can improve the local economy (Pereira et al., 2020). Yet, in most cases, water bodies are introduced with non-native fish species because the authorities envision short-term objectives, have poor scientific support and their assessments were biased towards personal interests (Agostinho et al., 2004, 2007, 2010). For instance, in the 1970s, the Puerto Rican aqriculture ministry was keen to rapidly improve the local economy. They encouraged locals to practice non-native fish culture since there was market demand for *Cichla* spp. (Neal et al., 2017). However, after several episodes of accidentally releasing *C. temensis* into the wild, the plunging of biodiversity in Puerto Rico brought negative impacts to the local fisheries economy (Bunkley-Williams et al., 1994; Bower et al., 2016; Neal et al., 2017; Figure 2).

While *C. temensis* and *C. ocellaris* were introduced into Florida and Texas (North America) whereas only *C. ocellaris* into Lake Gatun (Panama) after the 1967 damming projects, this action was convincing to be able to attract anglers, improve wild stock diversity and increase the inland capture fisheries earnings (Zaret and Paine, 1973; Rutledge and Lyongs, 1976; Howells and Garrett, 1992; Shafland and Stanford, 1999). With the objective achieved, *Cichla* thrived in the man-made lakes and became an attraction for tourism. Nevertheless, after 46 years, detrimental depletion of native species has occurred since *Cichla* extended into adjacent tributaries and expanded their range about 20 km from the initial introduction point (Zaret and Paine, 1973; Shafland, 1999b; Escobar-Camacho et al., 2019). Fortunately, the North American winter season is able to reduce *C. temensis* and *C. ocellaris* abundances since both species are unable to withstand extremely cold waters despite having already residing in these waters for several generations (Shafland et al., 2008).

#### 3.2.3 Sport-fishing

Sport-fishing is a community-based recreational activity that provides job opportunities and income in North America, several European countries and also in Brazil (Howells and Garrett, 1992; Shafland, 1999a; Cooke and Suski, 2004; Holley et al., 2008; Barroco et al., 2018; Golani et al., 2019). In Texas and Florida (North America) alone, sport-fishing is an activity participated by some 36 million anglers that collectively supports the fisheries and tourism industry with an annual revenue in exceed of USD 2.4 billion (Courtenay et al., 1973; Shafland and Stanford, 1999). In Brazil, sport-fishing has provided job opportunities to about 250,000 citizens and produced a turnover of USD 500 million (Barroco et al., 2018). Similarly, 16 million anglers contributed to over 150,000 metric tonnes of catch in Europe (Cowx, 2015). Perhaps the fast moving and aggressive nature of *Cichla* as claimed by the anglers in Tasik Telabak (Malaysia) are reasons for its translocation world wide (Figure 3). In addition, *C. ocellaris* is preferred by anglers because it is less vulnerable (<5 % mortality) to catch-and-release fishing (Figure 3; Shafland, 1999b; Shafland and Stanford, 1999; Cooke and Suski, 2004; Holley et al., 2008; Rahim et al., 2013; Barroco et al., 2018; Khaleel et al., 2020). In fact, only 97 fish were killed by inexperience hook release where ±2 % of the *C. ocellaris* deaths occurred after lethal hook puncture to the gills (Thomé-Souza et al., 2014; Bower et al., 2016).

#### 3.2.4 Aquaculture

Aquaculture produce has supported the fisheries sector since 1970s where *Oreochromis* sp. from Nile and Shire rivers (Africa) were introduced into Asia and Floriano (Brazil) for food security (Dey and Gupta, 2000; Kamal and Mair, 2005; Neves, 2008). Meanwhile, demands for *C. ocellaris* resulted to its introduction into aquaculture, pet trade and sport angling in Brazil (Perez et al., 2000). Unlike the *Oreochromis*, *Cichla* culture is prone to cannibalism because the growing fish cannot adapt to pellet feeding. Therefore, *Cichla* culturists developed their own interventions where only a handful of operators are successful to spearhead the culture of this fish during 1990–2008 (Moura et al., 2000; Cyrino and Kubitza, 2003; Britton and Orsi, 2012; Salaro et al., 2012, Figure 4).

**Figure 4:**
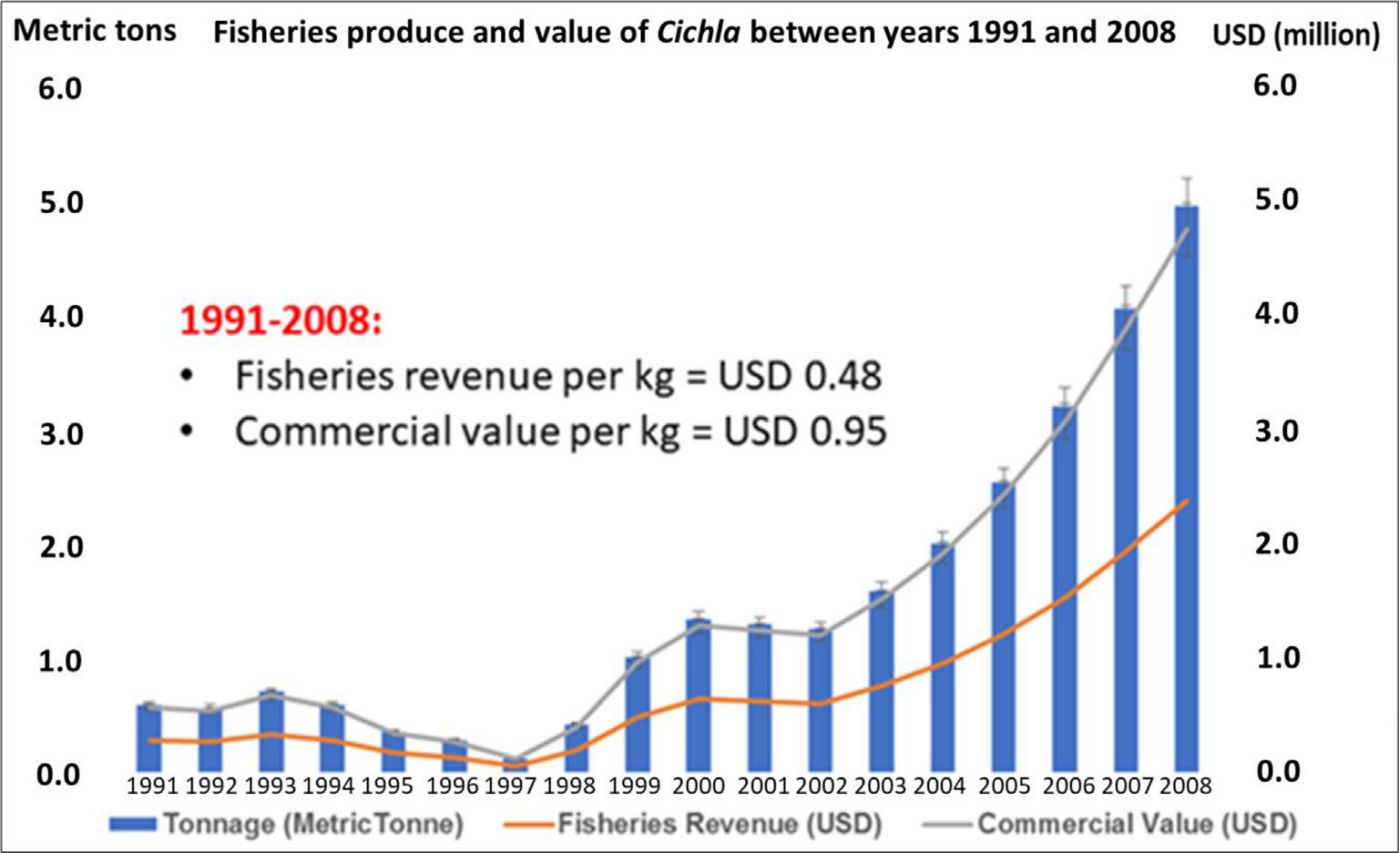
Global fisheries and commercial gains from peacock bass between 1991 and 2008.

Aquaculture has improved the genetics of cultivated animals by developing fast growing, environment tolerant and disease resistant varients (Saint-Paul, 2017). Along with poor management, ignorance, inadequate knowledge and floods, the unintentional release of safe and harmful species from aquaculture is liable for their availability in the wild (Courtenay and Robins, 1989; Daniel Carvalho et al., 2010; Azevedo-Santos et al., 2011; Vander et al., 2016). Interestingly, *Cichla* spp. is naturalized by decree in Brazil and Venezuela which means, this cichlid has heritage value, the wildstocks are protected using local laws and the fish in culture systems are important for local food security (Shafland and Standford, 1999; Economidis et al., 2000; Pelicice et al., 2014; Franco et al., 2018; Ota et al., 2019). Therefore, the release of genetically improved *Cichla* spp. from culture systems into the wild is not considered a crime by the local commissions in Brazil and Venezuela.

Such an implementation has permitted the interaction between wild and culture *Cichla* stocks where hybrids were emerging in Brazil, Peru and Guyana (Pelicice et al., 2014; Ota et al., 2019). *Cichla* spp. hybrids are improved versions of existing wildstocks which means, they are adapted to the environment, less susceptible to diseases and are geared to displace the weaker species. Overall, researchers around the world were observing the plunging of native fish populations within three years of culturing *Cichla* spp. (Zaret and Paine, 1973; Shafland, 1999b; Sultana and Hashim, 2015; Sharpe et al., 2017).

### 3.3 Rise of an emerging concern

Non-native species that are introduced in small numbers will assimilate, adapt and reproduce before occupying higher trophic positions in the food web (Chapman et al., 2016). Introduced *Cichla* spp. can tolerate dissolved oxygen (2.9–8.0 mg/L) and water temperatures (26–29.2 °C) of tropical environments while also experiencing 77 % rapidness for their growth rates and are able to produce offspring that are tolerant to similar conditions (Straškraba et al., 1993; Shafland, 1999a; Chellappa et al., 2003a, b; Espínola et al., 2014; Bower et al., 2016; Sharpe et al., 2017; Franco et al., 2018; Schofield et al., 2019).

While aforementioned studies show that brief durations (1–3 years) are sufficient for *Cichla* spp. to invade a water body, this lag-phase can also takes from years to decades depending on the threats, prey species and the size of the water body (Neal et al., 2006; Kovalenko et al., 2010; Aguiar-Santos et al., 2018). Yet, only transitional extreme weather (<0–38 °C) of summer and winter months is successful to prevent the widespread of culture-escape *Cichla* spp. in North America (Howells and Garrett, 1992). Since this cichlid is native to South America, mass culture during 1970–1980 is responsible for its availability throughout this region. Only after 45 years, *C. monoculus* and *C. kelberi* emerged to become a concern in Lake Gatun (Panama) (Sharpe et al., 2017) and Lajes Reservoir (Brazil) (Santos et al., 2019). Even the presence of predators like *Micropterus* sp. and equally invasive *Oreochromis* sp. were unable to threaten the survival of *Cichla* spp. that thrived in Corumbá, Paraná, Orinoco and Rio Negro – Guainía (Brazil) after they escaped from aquaculture pens (Simberloff and Stiling, 1996; Shafland, 1999b; Mack et al., 2000; Gomiero and Braga, 2004; Neal et al., 2006; Fugi et al., 2008; Pelicice and Agostinho, 2009; Valverde et al., 2019).

### 3.4 Spills from *Cichla* spp. introduction

At present, *Cichla* spp. is an emerging concern for its predatory behaviour. Unaware to many, this cichlid may also introduce disease causing microbes and parasites into new environments. While transitional weather influences fisheries distribution in river systems (Nelson et al., 2016a,b; Nelson et al., 2019b; Zauki et al., 2019a, b), it also affects the distribution and availability of microorganisms in the water body. It only takes one compatible intermediate host in oligotrophic waters for microorganisms like parasites to reproduce (sporulation, fission, budding and egg production) or continue with their life cycle (Hart and Reynolds, 2002; Levsen et al., 2008). For instance, *Dactylogyrus* sp. (monogenea or gill fluke) completes its life cycle in mouth- and substrate brooding cichlids and this makes *Cichla* spp. vulnerable to parasitism (Pouyaud et al., 2006; Šimková et al., 2006; Mendlová and Šimková, 2014; Vanhove et al., 2016; Paschoal et al., 2016; Jorissen et al., 2018). Also, an acanthor may use *Cichla* spp. to develop into a cystacanth but, later development into the acanthocephalan worm may not involve this cichlid (Nicholas, 1967; Ŝimková et al., 2006, Ŝimková et al., 2004; Mendlová, and Šimková, 2014). In short, *Cichla* spp. may be infected by monogenea, acanthocephala and blood fluke because these parasites settle on bottom susbrate when present without a host (Fuller, 2015; Ferreira-Sobrinho and Tavares-Dias, 2016; Luque et al., 2016; Paschoal et al., 2016; Januario et al., 2019, Table 3).

**Table 3:** A compilation of parasites associated to *Cichla* spp. in its native habitat.

Parasite category	Parasite species	Citations
Monogenea	*Gussevia alioides*	Kohn and Cohen, 1998
	*G. dispar*	Kohn and Cohen, 1998
	*G. tucunarense*	De Azevedo et al., 2007
	*G. arilla*	Yamada et al., 2009
	*G. longihaptor*	Yamada et al., 2011
	*G. undulata*	Delgado et al., 2012
	*G. disparoides*	Bittencourt et al., 2014
	*Sciadicleithrum umbilicus*	Kohn and Cohen, 1998
	*S. ergensi*	Yamada et al., 2011
	*S. uncinatum*	Yamada et al., 2011
Acanthocephala	*Quadrigyrus machadoi*	Yamada et al., 2011
	*Crassicutis cichlasomae*	Vanhove et al., 2016
Blood fluke	*Schistosoma mansoni*	De Marco Júnior, 1999

While parasite spillover from *C. kelberi* is witnessed for *Hyphessobrycon eques* in Rosana Lake (Brazil), the spillback of Tilapia Lake Virus onto *C. monoculus* occurs after its interaction with *Oreochromis* sp. (also a cichlid) in Timah Tasoh, Malaysia (Pelicice and Agostinho, 2009; Abdullah et al., 2018). From both observations, it is learnt that spillover of miroorganisms from *Cichla* spp. becomes an additional invasion mechanism for this cichlid and if not, the spillback of existing microbes may harm the introduced *Cichla* spp. (Carpenter et al., 1985; Estes, 1995; France et al.*,* 1998; Jennings et al., 2001; Woodward et al., 2002; Font, 2003; Jennings and Mackinson, 2003; Torchin et al., 2003; Layman et al.*,* 2005; Kelly et al., 2009; Sarmento, 2012; Blakeslee et al., 2013; Yamada and Takemoto, 2013; Frankel et al., 2015; Winnie and Creel, 2017).

### 3.5 Learning from invading *Cichla* spp.

Miscommunication is responsible for the increased entries in Global Invasive Species Database (GISD) of International Union for Conservation of Nature (IUCN) and therefore, the Agenda 2030 is becoming challenging to achieve (DasGupta et al., 2019). In addition, keywords such as alien, exotic, domesticated, naturalized, foreign, emerging, invasive, allochthonous, non-indigenous, a concern and pest are not only relevant to non-native species (Humair et al., 2014; Courchamp et al., 2017, Table 2) but, the number of synonyms are rising after each and every management failure. While management measures like poison baits (Simberloff and Stiling, 1996; Innes and Barker, 1999; Cory and Myers, 2000), electric fishing (Schofield et al., 2019) and intensive angling (Santos et al., 2019) are used to manage *Cichla* spp. overpopulation, it negatively impacted native *Cichla* spp. populations, threatened other non-target species or completely destroyed the habitat (Araújo et al., 2005; Simberloff, 2008; dos Santos et al., 2014; Santos et al., 2019). Rapid actions always produce fast results because it lacks surveillance and is briefly (sometimes one-off) implemented. Therefore, individuals who propose such actions are not interested in detection studies, long-term monitoring, identification of drivers and ecosystem functions simply because they commit to personal interests (Myers et al., 2000; Mack and Lonsdale, 2002; Leuven et al., 2017).

Every country introduced with *Cichla* spp. were developing taskforce to control its widespread in affected water bodies but, their actions were delivered to the entire area rather than isolating the target from the non-target species (Epanchin-Niell and Wilen, 2012; Büyüktahtakın and Haight, 2018; Bonneau et al., 2019). In fact, several decisions that favoured revenue over existing biodiversity (Meiners-Mandujano et al., 2019; Ramírez-Albores et al., 2019), were challenged by priorities (Pelicice et al., 2014; Ota et al., 2019), work ethics, hiring schemes, conflicting interests (Fischer et al., 2014; Humair et al., 2014; Pietrzyk-Kaszyńska and Grodzińska-Jurczak, 2015) divided tradition and total neglect on local knowledge (Fischer et al., 2014; Humair et al., 2014). All of these decisions are developed from top-down management that prioritize on theory and book-based scientific evidence (Humair et al., 2014). In short, the success of including local knowledge into fisheries practices also extends benefits to other species and the environment (*c.f.* Zauki et al., 2019a, b) which means, the management of *Cichla* spp. should consider community opinions because they constantly interact with the animal.

### 3.6 An improvised management plan

Communication on invading fish species like *Cichla* spp. is possible through digital media, signages and brochures (Schofield et al., 2019; Shackleton et al., 2019). Considering awareness as a form of communication with the public, it also promotes species detection while informing about impacts brought by the invader (Neal et al., 2006; Gallardo et al., 2016; Liao et al., 2019). Yet, public opinions should not be a literal account to manage a species with emerging cencerns because introduced species have a changed population dynamics, relationship with the environment and could appear as hybrids (Courchamp et al., 2017; Jaffar et al., 2019). Considering multidisciplinary (engineers, mathematicians, accountants, and underwriters) the frontier for decision making, this team uses ecological re-engineering and green accounting to assess damages done by an invading species (Hasting et al., 2006; Olson, 2006; Blackburn et al., 2011; Epanchin-Niell and Hastings, 2012).

**Figure 5:**
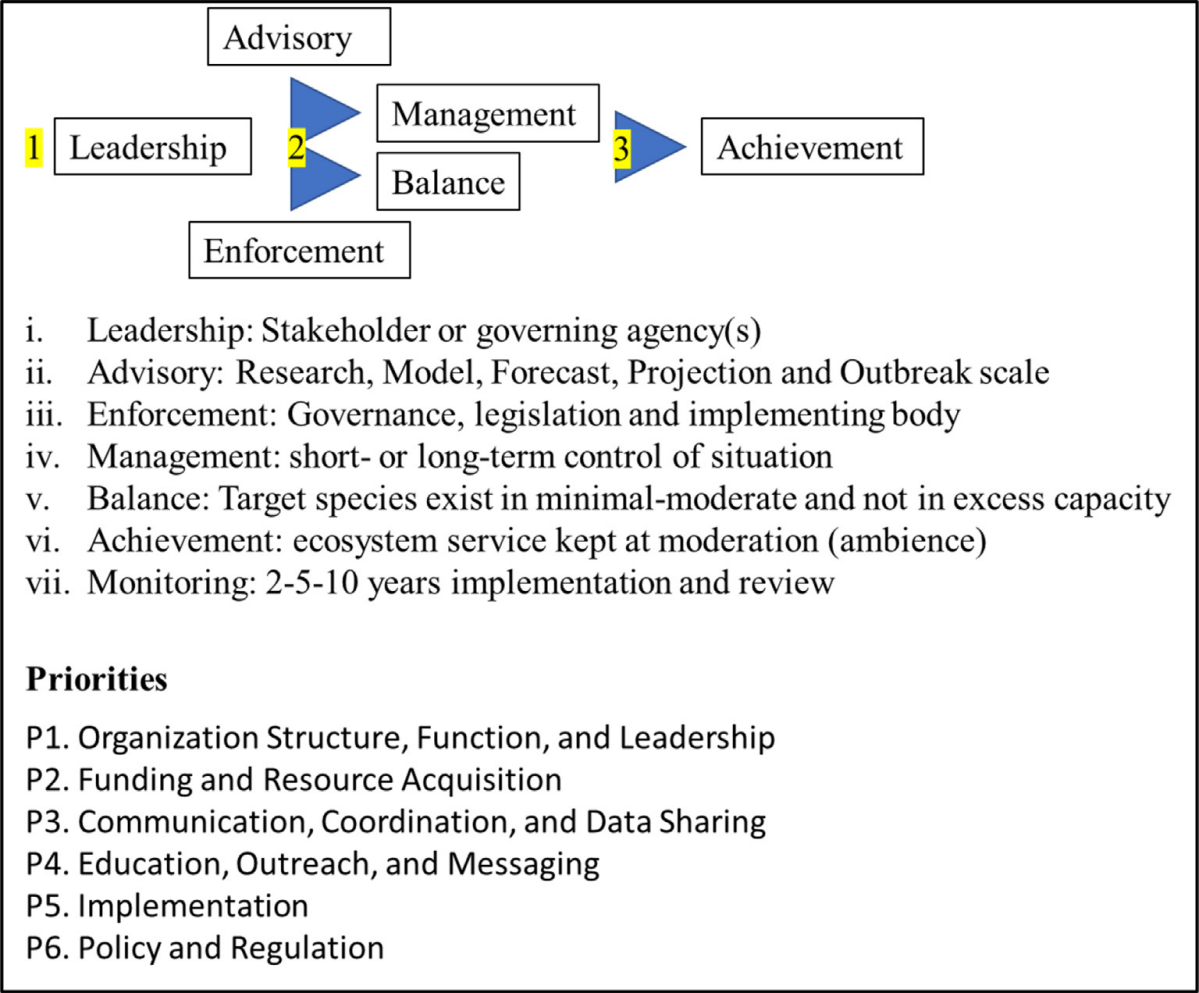
Framework containing action and priorities for species with emerging concern.

In addition, the creation of machine-learning platforms that combine bottom-up and top-down opinions (geographical focus, habitat, and taxonomic data) is able to scale every decision with a rate so that authorities can choose the most effective approach (Olden et al., 2002; Dana et al., 2014; Büyüktahtakın and Haight, 2018; Schofield et al., 2019, Figure 5). For instance, information from the digestive tract (stomach contents, faecal or scat) and predation (carcasses, injury, eggs and prey-predator proportions) can produce a dietary calender and the addition of climate data can establish prey-predator relationships, identify reproduction seasons and predict growth durations (*c.f.* Calver et al., 1998; Brown and Sherley, 2002; Park, 2004; Fugi et al., 2008; Schofield et al., 2019). On the contrary, researchers often neclect on zone definitions and its scale. For instance, translocation of *Cichla* spp. is permitted in zones with severe invasion but, the fish must be euthanized (and dismembered) before its movement within zones that are free from this species. By far, successful management can only be accomplished by collaboration (engagements, training and education) where decision-makers and communities work together to form an understanding. The breach of this understanding has a scale of (legislation) that also require compensations (fines and punishments) (Figure 5). This should follow by monitoring and reassessments (remain in IUCN Red List or shifted into the Green List) so that actions to recover a species does not neglect another species (Courchamp and Caut, 2006; Genovesi and Carnevali, 2011, Figure 5). For instance, introduction of native species after *Cichla* spp. removal should follow with a series (2-5-10 years) of assessments (and review of action plan) so that early signs of local invasion is revealed (van Vuuren et al., 2007; Courchamp et al., 2011; Kok et al., 2017; DasGupta et al., 2019; Schofield et al., 2019). Overall, a management plan that contains different levels of actions for invading species (inform, gather, compile, communicate and re-assess; Figure 5) is already integrated with sustainable indicators of Agenda 2030 and the Aichi targets in Convention of Biological Diversity 2016 and does not require additional review on its definitions.

## 4 Conclusions and recommendations

This article communicates on *Cichla* spp. as a non-native species with potential to become an emerging concern in North America, Africa and Asia. We learnt that 15 species of *Cichla* exists and they are distinguished by 3–5 horizontal band marks along with gold outline black anulus where only *C. kelberi, C. monoculus, C. ocellaris* and *C. temensis* were sought for sport fishing, aquarium keeping and aquaculture. Also, *Cichla* spp. requires 11–12 months to mature in which the larvae and juvenile adopt mixtroph diets whereas the sub- and mature-adults are true piscivore. The ability of *Cichla* spp. to cause active (predation) and passive (parasite and disease spillover) invasion is limited to environment settings and underlying threats. Implementations such as poison bait, electric fishing and intensive angling have been used over the years to suppress *Cichla* spp. overpopulation. However, these implentations were unreliable because decision are made by authorities that do not understand the biology of *Cichla* spp. after becoming introduced into tropical and sub-tropical regions. Therefore, we utilize secondary data to map *Cichla* spp. availability outside their native geographies while highlighting weaknesses such as top-down, short-term, conflicting interest and poor decision making as management failures. We recommend an updated management strategy that uses measures to detect, acquire, execute and track resources after taking into consideration ‘crowd wisdom’, horizontal screening and machine learning for short- and long-term strategies which need to be reviewed in a sequence of 2, 5 and 10 years. Additional suggestions include:

I. Develop knowledge on genetic or protein manipulation that produces defected *Cichla.* Defected *Cichla* spp. (second-generation filial, F2) are either, intolerant to the environment or sterile.
II. Introduction schemes for *Cichla* spp. must be regulated by a local legislation where aquarium dumping or active release of this fish into the wild is regarded an offence and punishable by law.
III. Future studies should be aligned with the SDGs (Agenda 2030) and membered by experts from various fields, education, and occupation.
IV. Current local, regional, and international databases on successful eradication or otherwise should be developed and disseminated among the researchers, authorities and policymakers.
V. The annual monitoring of an introduced species is an effective way of early invader detection. Zoning must be implemented to control their movement.
VI. Engaging the public with invasive species management gathers opinions from crowds whereby this form of transparency convinces the public that actions are measurable and have an outcome.
VII. Conservation and economic gains should be themed with sustainability so that resource governance contains compensation schemes for communities whose livelihood becomes vulnerable after such impacts.

## Declarations

### Author contribution statement

All authors listed have significantly contributed to the development and the writing of this article.

### Funding statement

This research did not receive any specific grant from funding agencies in the public, commercial, or not-for-profit sectors.

### Declaration of interests statement

The authors declare no conflict of interest.

### Additional information

No additional information is available for this paper.
